# The Development of Intensive Care Unit Acquired Hypernatremia Is Not Explained by Sodium Overload or Water Deficit: A Retrospective Cohort Study on Water Balance and Sodium Handling

**DOI:** 10.1155/2016/9571583

**Published:** 2016-09-14

**Authors:** M. C. O. van IJzendoorn, H. Buter, W. P. Kingma, G. J. Navis, E. C. Boerma

**Affiliations:** ^1^Department of Intensive Care, Medical Centre Leeuwarden, P.O. Box 888, 8901 BK Leeuwarden, Netherlands; ^2^Department of Internal Medicine, University Medical Centre Groningen, P.O. Box 30001, 9700 RB Groningen, Netherlands

## Abstract

*Background*. ICU acquired hypernatremia (IAH, serum sodium concentration (sNa) ≥ 143 mmol/L) is mainly considered iatrogenic, induced by sodium overload and water deficit. Main goal of the current paper was to answer the following questions: Can the development of IAH indeed be explained by sodium intake and water balance? Or can it be explained by renal cation excretion?* Methods.* Two retrospective studies were conducted: a balance study in 97 ICU patients with and without IAH and a survey on renal cation excretion in 115 patients with IAH.* Results.* Sodium intake within the first 48 hours of ICU admission was 12.5 [9.3–17.5] g in patients without IAH (*n* = 50) and 15.8 [9–21.9] g in patients with IAH (*n* = 47), *p* = 0.13. Fluid balance was 2.3 [1–3.7] L and 2.5 [0.8–4.2] L, respectively, *p* = 0.77. Urine cation excretion (urine Na + K) was < sNa in 99 out of 115 patients with IAH. Severity of illness was the only independent variable predicting development of IAH and low cation excretion, respectively.* Conclusion*. IAH is not explained by sodium intake or fluid balance. Patients with IAH are characterized by low urine cation excretion, despite positive fluid balances. The current paradigm does not seem to explain IAH to the full extent and warrants further studies on sodium handling in ICU patients.

## 1. Introduction

ICU acquired hypernatremia (IAH), defined as a serum sodium concentration (sNa) of more than 145 mmol/L, is a regularly occurring condition in a large variety of intensive care patients [1]. In previous publications, the incidence of IAH varies from 3 to 17% [2–5]. We previously reported an incidence IAH between 6% and 9% [6]. In several studies, IAH was associated with higher morbidity and mortality and a prolonged length of stay in the ICU [4, 5, 7–9]. Moreover, recent observations by Darmon et al. confirmed the association between IAH and mortality with an even lower cutoff value for sNa ≥ 143 mmol/L [7].

Under normal circumstances, sNa is maintained within relatively narrow limits by osmo- and volume-regulation. A change in sodium balance is associated with only subtle changes in sNa [10, 11]. Theoretically, hypernatremia is caused by a disturbance in water homeostasis and sodium content [12–16]. These mechanisms are derived from the Edelman equation, which in simplified form is as follows [17]:

(1) $$\begin{array}{c} {\left[{Na}^{+}\right]=\frac{\left(\text{Total exchangeable }{Na}^{+}+\text{total excangeable }K^{+}\right)}{\text{Total body water}}.}^{\;} \end{array}$$

In the past decades, IAH is mainly seen as an iatrogenic complication. On the one hand, excessive sodium intake during critical illness, attributed to the infusion of sodium-rich fluids, may play a role [12, 14, 18–20]. On the other hand, decrease in total body water, caused by renal or extrarenal water loss, or insufficient water intake may enhance the rise in sNa. ICU patients either are incapable of swallowing or have limited access to free water whilst being sedated during mechanical ventilation [7, 14]. Excessive water loss can be due to diabetes insipidus, the use of diuretics, osmotic diuresis (e.g., in case of high urea excretion), electrolyte disorders, increased or nonreplenished insensible loss, nasogastric suction, or fluid loss via tubes or drains [12, 19]. Healthy individuals, subject to intravenous sodium loading, display increased renal sodium excretion to maintain homeostasis [21–23]. In critically ill patients, an impaired ability to excrete cations has been reported, independently of their volume status [14, 15]. This is in line with our own observations that consistent reduction of sodium intake, by replacement of all sodium-rich resuscitation fluids, did not seem to change the overall incidence of IAH in our own ICU department [6].

As a first step to unravel the aetiology of IAH, we performed two complementary observational studies to answer the following questions: First, can the development of IAH be (fully) explained by parameters of sodium intake and water balance? Or could it be explained by renal cation excretion?

## 2. Methods

### 2.1. Patients and Setting

This study consisted of two complementary parts: one balance study and another on renal cation excretion. The balance study was a single-centre retrospective cohort analysis in patients admitted to the ICU from September 2013 until February 2014. The ICU is a 22-bed combined medical and surgical unit in a tertiary teaching hospital. All patients with a length of stay (LOS) in the ICU ≥ 48 hours were included. Exclusion criteria were sNa ≥ 143 mmol/L on admission and renal replacement therapy. Patients were divided into two subgroups: one group of patients that developed a sNa ≥ 143 mmol/L and one group that did not. An alternative sNa ≥ 145 mmol/L cutoff value was also predefined for secondary analysis.

Simultaneously, a single-centre cohort analysis on renal cation excretion was performed. As a by-product of an ongoing trial, spot urine samples were available in patients with IAH. These samples were obtained as soon as possible after the occurrence of IAH. Inclusion criteria for this study were IAH and a LOS ICU ≥ 48 hours. Exclusion criteria were sNa ≥ 143 mmol/L on admission and renal replacement therapy. Spot urine samples were collected in the period between September 2013 and April 2015 and retrospectively analysed. Groups were classified on the assumption that in nonhypovolemic patients a total renal excretion of sodium and potassium lower than sNa implies impaired ability of the kidney to excrete cations [15]. In group 1, total renal cation excretion (urine (uNa) + urine potassium (uK)) was < sNa. In group 2, total renal cation was ≥ sNa.

### 2.2. Data Collection

Data were extracted from the patient data management system (PDMS). The following patient characteristics were identified: gender, age, Acute Physiology and Chronic Health Evaluation (APACHE) IV-score on admission [24], daily Sequential Organ Failure Assessment (SOFA) scores [25], reason for admission, and length of ICU stay. Routine daily collected measurements of sNa, serum creatinine concentration, and serum urea concentration were used. sNa was measured with point-of-care-testing (POCT, ABL800 AutoCheck®, Radiometer Pacific Pty. Ltd., Australia and New Zealand). In addition, registration of total sodium intake (including enteral and parenteral feeding, administered fluids, and sodium content of administered drugs and their solvents), fluid balance (derived from PDMS minus 500 mL anticipated insensible loss/day), diuresis, and administration of diuretics were part of daily routine. Urine cation excretion was calculated as the sum of urine sodium and potassium concentrations derived from a spot urine sample. A local ethics board (Regionale Toetsingscommissie Patiëntgebonden Onderzoek, Leeuwarden, Netherlands) waived the need for informed consent, according to applicable laws.

### 2.3. Statistical Analysis

Data were collected in and analysed with SPSS 20 (IBM, New York, USA). Distribution of data was evaluated by histograms and Shapiro-Wilk testing. Data are expressed as median with interquartile range (IQR) or as a number with the corresponding percentage.

In the balance study, sNa was used as a dichotomous variable to determine the difference in total sodium intake and fluid balance between groups after 24 and 48 hours. Applicable tests for independent variables were conducted to compare groups. Outcomes were considered significant at *p* ≤ 0.05. Backwards multivariate logistic regression analysis was performed, including all variables with a *p* value ≤ 0.25 in the univariate analysis. In case of categorical variables, the first category served as reference. Probability for stepwise entry and removal were set at 0.05. Outcomes are expressed as odds ratio (OR) with a confidence interval (CI) of 95%.

## 3. Results

### 3.1. Balance Study

During the study period, 97 patients were eligible for inclusion. 47 patients were included in the IAH group (sNa ≥ 143 mmol/L) and 50 patients in the non-IAH group (sNa < 143 mmol/L).

Baseline characteristics are presented in Table 1. Apart from severity of illness scores, which were higher in patients developing IAH, there was no significant difference between groups at baseline.

**Figure 1:**
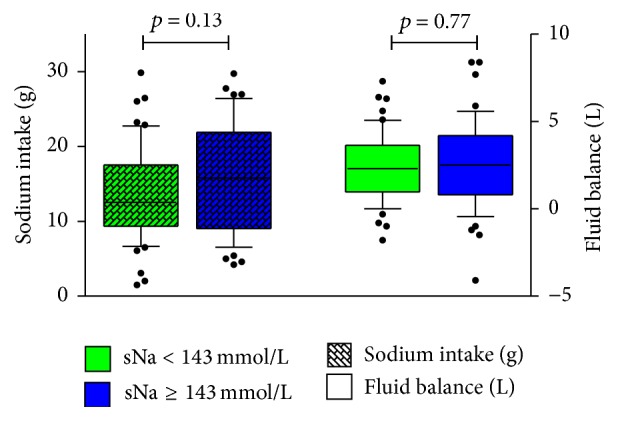
Total sodium intake and fluid balance 48 hours after admission in patients with and without developing IAH. sNa: serum sodium concentration.

Median number of days until fulfilment of the IAH-criterion was 3 [2–4]; median duration of sNa ≥ 143 mmol/L was 3 days [1–9]. Total sodium intake after 48 hours was 12.5 [9.3–17.5] grams in the non-IAH group versus 15.8 [9–21.9] grams in the IAH group, *p* = 0.13. Fluid balances were positive in both groups and did not differ between groups at 24 and 48 hours after admission. Central venous pressure, as an indirect parameter of volume status, did not differ between groups (Tables 2 and 3 and Figure 1). Spot urine samples were available from 22 patients with IAH. Median amount of sodium in these samples was 45 mmol/L [10–94].

Length of stay of patients with IAH was significantly longer in comparison to the control group (4 [3–5] versus 6 [4–12], *p* < 0.001, Table 2). In a multivariate logistic regression analysis, severity of illness, defined by APACHE IV-scores, remained as the only significant factor in the development of IAH (OR 1.020 (CI 1.004–1.035), *p* = 0.01). Analysing data with sNa ≥ 145 mmol/L as an alternative cutoff value for IAH did not significantly change outcomes. These data are provided in the electronic supplemental material (ESM) available online at http://dx.doi.org/10.1155/2016/9571583.

### 3.2. Renal Cation Excretion Study

Renal cation excretion was measured in 115 patients with IAH. 99 patients were included in the group with low cation excretion (uNa + uK < sNa) and 16 patients in the group with high cation excretion (uNa + uK ≥ sNa). Baseline characteristics are provided in Table 4. At the time of urine analysis, median sNa in group 1 was 144 [143–147] mmol/L versus 145 [143–146] mmol/L in group 2 (*p* = 0.85). Median sodium excretion was 38 [15–67] mmol/L in group 1 and 133 [104–152] mmol/L in group 2 (*p* < 0.001). Potassium excretion was also significantly lower in group 1 (36 mmol/L versus 45 mmol/L, *p* < 0.001). In a multivariate logistic regression model, APACHE IV remained the only significant independent predictive variable for low urine cation excretion.

## 4. Discussion

The balance study showed that development of IAH is not fully explained by differences in sodium intake or fluid balance. Our data do not seem to be completely in line with previous literature and with the equation as described by Edelman. Over the last decades, the common opinion has been that IAH is a primary iatrogenic problem caused by either sodium overload, lack of adequate water intake, or a combination [1, 12, 14, 16, 18–20, 26–28]. However original data on the differences in sodium intake and fluid balance between ICU patients with and without IAH seem to be scarce. In addition, some authors have focused on specific sources of sodium intake, such as resuscitation fluids or line flushing [18]. Our PDMS provided us the opportunity to incorporate all sources of sodium intake, including tube feeding and medication. In addition, populations investigated in previous publications were considerably smaller than in our study [20, 26]. Lastly, an important difference between this study and previous publications is the cutoff value for IAH. We deliberately chose 143 mmol/L as cutoff value since Darmon et al. demonstrated the potential detrimental effects of even mildly elevated sNa in critically ill patients [7]. In previous studies, a cutoff value of 145 mmol/L or even 150 mmol/L was not uncommon [1, 12, 14, 18–20, 26–28].

This reflects not only the change in mindset with respect to the relevance of IAH, but also the focus on the reduction of excessive sodium intake due to fluid overload and fluid composition in comparison to previous literature. It is conceivable that in previous publications the widespread use of “isotonic” saline in combination with more liberal infusion triggers has been a contributing factor in the development of IAH [12]. However, even in our setting, with tight infusion triggers and lower sodium content of resuscitation fluids, median sodium intake is far beyond the recommended daily amount of 2.6 g sodium and a specific group of ICU patients still develops IAH [6]. This suggests both differences in sodium handling between patients that do and do not develop hypernatremia and the potential for other contributing factors in the development of IAH not yet identified.

The study on renal cation excretion revealed that most patients with IAH seem to have an impairment in renal cation excretion. Such inability to excrete cations was previously suggested by others as a contributing factor in the aetiology of IAH [14–16, 26, 27]. Indeed, in our study on renal cation excretion, the vast majority of patients with IAH displayed a total renal cation excretion below serum sodium concentration. This is unlikely due to a water deficit, since fluid balances were clearly positive. Strictly, this does not rule out an absolute water deficit but makes it unlikely to be the only contributing factor. Suggested mechanisms are tubular dysfunction in the cause of acute renal failure or osmotic diuresis as a result of enhanced urea excretion [16, 26, 29]. Although we did not measure urea excretion, the positive fluid balances in our patients make excessive renal water loss by osmotic diuresis as a cause of IAH unlikely.

If IAH cannot be explained by sodium intake or fluid balance, the issue of an alternative explanation arises. The fact that the APACHE IV-score, as markers of severity of illness, was independently associated as risk factor for IAH in the balance study and for low renal cation excretion, respectively, fuels the idea of a more complex aetiology of IAH. Such alternative explanation could be found in a third compartment for storage of sodium. Already in 1910 Padtberg mentioned this compartment [30]. Storage of osmotically inactive sodium in (extremely) high concentrations has been reported in cartilage, muscle, bone, and skin [31–33]. In healthy volunteers, water-free sodium storage has been described [32]. In recent papers, attention to this compartment was renewed with focus on hypertension and its treatment [34–36]. In animal and in vitro models, differences in sodium storage capacity were found and appeared to be related to the development of hypertension [34, 36]. Binding of sodium to proteoglycans seems to be the major mechanism for intracutaneous nonosmotic sodium storage and thereby serve as a conceivable third compartment. Altered configuration with consequent changes in electrical binding capacity has been suggested during inflammation [37]. Our observation that IAH was related to severity of illness, independent of sodium intake and fluid balance, may be in line with an inflammation mediated pathway. Further investigations on these mechanisms in relation to IAH should be initiated.

## 5. Limitations of the Study

Due to the retrospective single-centre design, this study has its limitations. Full fluid and sodium balances were not performed; sodium and water content in sweat and stool were left out of the equation. In this study, insensible loss of 500 mL per day was estimated [38, 39]. Urine analysis was limited to spot urine samples and was restricted to patients with IAH. ADH-concentrations, urine urea concentrations, and urine osmolality were not measured. Mentioned fluid balances did not include fluid administration prior to ICU admission. Due to diurnal variation in renal sodium excretion spot urine samples are not optimal in evaluating urine sodium excretion.

## 6. Conclusion

In spite of the current opinion, development of IAH is not (fully) explained by sodium intake or fluid balance. This lack of association between IAH and sodium intake and/or fluid balance suggests other factors unaccounted for in the current paradigm. Thereby, IAH does not seem to be a primary iatrogenic complication. Severity of illness as an independent risk factor for both IAH and low renal sodium excretion may reflect other contributing factors, including sodium handling in the third compartment, not yet identified. Therefore, prospective studies concerning handling and distribution of sodium and sodium balance, including hormone activity, to unravel the complex aetiology of IAH are needed.

## Supplementary Material

With a cut-off value of 145 mmol/L for IAH variables concerning severity of illness remained significantly different between groups, with patients that developed IAH as the more severe I'll group. Sodium intake after 48 hours reached a significant difference, however still with high amounts of sodium intake in both groups.

## Figures and Tables

**Table 1:** Baseline characteristics balance study. sNa: serum sodium concentration.

	s[Na] < 143 mmol/L	s[Na] ≥ 143 mmol/L	*p* value
Number of patients, *n* (%)	50 (51)	47 (49)	
Male gender, *n* (%)	34 (68)	29 (62)	0.53
Age, years	66 [61–73]	67 [57–77]	0.57
APACHE IV-score	58 [44–77]	68 [56–101]	0.01
SOFA score on admission	6 [4–8]	7 [4–10]	0.16
Reason for admission, *n* (%)			
Cardiovascular surgery	25 (50)	14 (30)	0.07
Sepsis	4 (8)	7 (15)	
Elective surgery	3 (6)	2 (4)	
Emergency surgery	10 (20)	5 (11)	
Cardiopulmonary	4 (8)	8 (17)	
resuscitation			
Miscellaneous	4 (8)	11 (23)	
Serum sodium on admission, mmol/L	138 [136–140]	138 [136–140]	0.25
Serum creatinine on admission, *µ*mol/L	93 [71–117]	85 [69–113]	0.55
Serum urea on admission, mmol/L	7 [5–7]	6 [5–8]	0.44

APACHE: Acute Physiology and Chronic Health Evaluation; SOFA: Sequential Organ Failure Assessment. Data are presented as median [IQR] or as absolute numbers (%).

**Table 2:** Main results balance study.

	s[Na] < 143 mmol/L	s[Na] ≥ 143 mmol/L	*p* value
Length of stay, days	4 [3–5]	7 [4–15]	<0.001
SOFA score after 24 hours	6 [4–7]	8 [5–10]	0.02
SOFA score after 48 hours	5 [3–6]	7 [4–10]	<0.001
Fluid intake after 24 hours, L	4.4 [3.7–5.6]	3.8 [2.9–6.3]	0.54
Fluid intake after 48 hours, L	7.5 [6–9.2]	6.9 [5.3–9.2]	0.59
Fluid balance after 24 hours, L^1^	2 [1–2.8]	1.6 [0.6–3.7]	0.78
Fluid balance after 48 hours, L^1^	2.3 [1–3.7]	2.5 [0.8–4.2]	0.77
Sodium intake after 24 hours, grams	9.6 [6.9–11.8]	9.7 [5.9–15.8]	0.70
Sodium intake after 48 hours, grams	12.5 [9.3–17.5]	15.8 [9–21.9]	0.13
Serum creatinine after 24 hours, *µ*mol/L	87 [66–130]	81 [65–110]	0.40
Serum creatinine after 48 hours, *µ*mol/L	79 [60–116]	77 [61–121]	0.91
Serum urea after 24 hours, mmol/L	8 [6–10]	7 [5–11]	0.47
Serum urea after 48 hours, mmol/L	8 [6–12]	9 [5–13]	0.71
Number of patients on furosemide after 24 h	5	4	1
Total dose furosemide after 24 h, mg	20 [20–60]	60 [25–400]	0.29
Number of patients on furosemide after 48 h	18	15	0.83
Total dose furosemide after 48 h, mg	30 [20–60]	40 [20–60]	0.19

sNa: serum sodium concentration; SOFA: Sequential Organ Failure Assessment. ^1^Fluid balances are as extracted from the patient data management system, minus 500 mL of expected insensible loss per day of admission. Data are presented as median [IQR] or as absolute numbers (%).

**Table 3:** Central venous pressure.

		s[Na] < 143 mmol/L	s[Na] ≥ 143 mmol/L	*p* value
CVP admission, mmHg	MV (*n* = 70)	10 [8–11]	11 [9–12]	0.05
	No MV (*n* = 7)	*n* = 2	*n* = 5	NA

CVP 24 hours, mmHg	MV (*n* = 45)	8 [5–11]	9 [5–12]	0.78
	No MV (*n* = 32)	6 [4–9]	5 [2–8]	0.20

CVP 48 hours, mmHg	MV (*n* = 30)	7 [3–11]	9 [6–12]	0.40
	No MV (*n* = 47)	5 [2–8]	6 [2–9]	0.58

CVP: central venous pressure; MV: mechanical ventilation; NA: not applicable.

**Table 4:** Baseline characteristics renal cation excretion study.

	Group 1 (uNa + uK < sNa)	Group 2 (uNa + uK ≥ sNa)	*p* value
Number of patients, *n* (%)	99 (86)	16 (14)	
Male gender, *n* (%)	74 (75)	9 (56)	0.14
Age, years	67 [57–74]	63 [42–70]	0.44
APACHE IV-score	88 [68–116]	62 [51–80]	0.02
SOFA score on admission	8 [7–11]	7 [5–9]	0.26
Reason for admission, *n* (%)			
Cardiovascular surgery	18 (18)	2 (12)	0.74
Sepsis	33 (34)	6 (38)	
Elective surgery	6 (6)	3 (19)	
Emergency surgery	5 (5)	0 (0)	
Cardiopulmonary	12 (12)	0 (0)	
resuscitation			
Miscellaneous	25 (25)	5 (31)	
Serum sodium on admission, mmol/L	137 [135–139]	139 [136–141]	0.16
Serum creatinine on admission, *µ*mol/L	94 [79–129]	78 [72–105]	0.22
Serum urea on admission, mmol/L	8 [6–12]	7 [5–8]	0.05

uNa: urine sodium concentration; uK: urine potassium concentration; sNa: serum sodium concentration; APACHE: Acute Physiology and Chronic Health Evaluation; SOFA: Sequential Organ Failure Assessment. Data are presented as median [IQR] or as absolute numbers (%).
