# Supplementary material for: The Development of Intensive Care Unit Acquired Hypernatremia Is Not Explained by Sodium Overload or Water Deficit: A Retrospective Cohort Study on Water Balance and Sodium Handling
Source: Crit Care Res Pract. 2016 Sep 14;2016:9571583. doi: 10.1155/2016/9571583 (PMC5040124; doi:10.1155/2016/9571583)
Supplement: Supplementary file 1 — With a cut-off value of 145 mmol/L for IAH variables concerning severity of illness remained significantly different between groups, with patients that developed IAH as the more severe I'll group. Sodium intake after 48 hours reached a significant difference, however still with high amounts of sodium intake in both groups. [file 9571583.f1.doc]

**Supplemental material**

|  | **s[Na] < 145mmol/l** | **s[Na] ≥ 145mmol/l** | **P-value** |
| --- | --- | --- | --- |
| Number of patients, n (%)  Male gender, n (%)  Age, years  APACHE IV  SOFA-score  Reason for admission, n (%)  Cardiovascular surgery  Sepsis  Elective surgery  Emergency surgery  Cardiopulmonary resuscitation  Miscellaneous  Serum sodium on admission, mmol/l  Serum creatinine on admission, µmol/l  Serum urea on admission, mmol/l | 66 (68)  42 (64)  66 [61-73]  59 [47-78]  6 [4-6]  30 (45)  6 (9)  4 (6)  13 (20)  8 (12)  5 (8)  138 [136-140]  92 [71-113]  7 [5-7] | 31 (32)  21 (68)  66 [57-77]  75 [60-98]  8 [5-10]  9 (29)  5 (16)  1 (3)  2 (7)  4 (13)  10 (32)  138 [135-140]  81 [67-122]  6 [4-8] | 0.82  0.93  0.02  0.03  0.02  0.85  0.68  0.61 |

**Table I:** Baseline characteristics, balance study. APACHE Acute Physiology And Chronic Health evaluation, SOFA Sequential Organ Failure Assessment. Data are presented as median [IQR] or as absolute numbers (%).

|  | **s[Na] < 145mmol/l** | **s[Na] ≥ 145mmol/l** | **P-value** |
| --- | --- | --- | --- |
| Length of stay, days  SOFA-score after 24 hours  SOFA-score after 48 hours  Fluid balance after 24 hours, L^1^  Fluid balance after 48 hours, L^1^  Administered sodium after 24 hours,   grams  Administered sodium after 48 hours,   grams  Serum creatinine after 24 hours, µmol/l  Serum creatinine after 48 hours, µmol/l  Serum urea after 24 hours, mmol/l  Serum urea after 48 hours, mmol/l  No of patients on furosemide after 24h  Total dose furosemide after 24h, mg  No of patients on furosemide after 48 h  Total dose furosemide after 48h, mg | 4 [3-5]  6 [4-8]  5 [3-7]  1.9 [0.9-2.8]  2.4 [1-3.7]  9.3 [6.8-12.1]  12.5 [9.2-17.7]  84 [66-115]  76 [59-112]  8 [6-9]  8 [6-12]  5 (8)  20 [20-60]  21 (32)  40 [20-60] | 10 [6-20]  8 [6-10]  8 [5-11]  1.9 [0.5-3.7]  2.6 [0.2-4.9]  11.6 [5.7-15.9]  15.9 [9.8-23.1]  94 [68-130]  85 [61-147]  8 [5-11]  9 [7-14]  4 (13)  60 [25-395]  12 (39)  50 [20-75] | 0.00  0.00  0.00  0.96  0.99  0.26  0.03  0.52  0.34  0.74  0.20  0.46  0.29  0.50  0.31 |

**Table II:** Results of investigated parameters, balance study. 1. Fluid balances are as extracted from the PDMS, minus 500ml of expected insensible loss per day of admission.
